# *CaHSL1* Acts as a Positive Regulator of Pepper Thermotolerance Under High Humidity and Is Transcriptionally Modulated by CaWRKY40

**DOI:** 10.3389/fpls.2018.01802

**Published:** 2018-12-07

**Authors:** Deyi Guan, Feng Yang, Xiaoqin Xia, Yuanyuan Shi, Sheng Yang, Wei Cheng, Shuilin He

**Affiliations:** ^1^Fujian Provincial Key Laboratory of Applied Genetics, Fujian Agriculture and Forestry University, Fuzhou, China; ^2^Key Laboratory of Plant Genetic Improvement and Comprehensive Utilization, Ministry of Education, Fujian Agriculture and Forestry University, Fuzhou, China; ^3^College of Crop Science, Fujian Agriculture and Forestry University, Fuzhou, China

**Keywords:** *Capsicum annuum*, *CaHSL1*, CaWRKY40, thermotolerance, positive regulator

## Abstract

Pepper (*Capsicum annuum*) is an economically important vegetable and heat stress can severely impair pepper growth, development, and productivity. The molecular mechanisms underlying pepper thermotolerance are therefore important to understand but remain elusive. In the present study, we characterized the function of *CaHSL1*, encoding a HAESA-LIKE (HSL) receptor-like protein kinase (RLK), during the response of pepper to high temperature and high humidity (HTHH). CaHSL1 exhibits the typical structural features of an arginine-aspartate RLK. Transient overexpression of CaHSL1 in the mesophyll cells of *Nicotiana benthamiana* showed that CaHSL1 localizes throughout the cell, including the plasma membrane, cytoplasm, and the nucleus. *CaHSL1* was significantly upregulated by HTHH or the exogenous application of abscisic acid but not by *R. solanacearum* inoculation. However, *CaHSL1* was downregulated by exogenously applied salicylic acid, methyl jasmonate, or ethephon. Silencing of *CaHSL1* by virus-induced gene silencing significantly was reduced tolerance to HTHH and downregulated transcript levels of an associated gene *CaHSP24*. In contrast, transient overexpression of *CaHSL1* enhanced the transcript abundance of *CaHSP24* and increased tolerance to HTHH, as manifested by enhanced optimal/maximal photochemical efficiency of photosystem II in the dark (Fv/Fm) and actual photochemical efficiency of photosystem II in the light. In addition, CaWRKY40 targeted the promoter of *CaHSL1* and induced transcription during HTHH but not in response to *R. solanacearum*. All of these results suggest that *CaHSL1* is directly modulated at the transcriptional level by CaWRKY40 and functions as a positive regulator in the response of pepper to HTHH.

## Introduction

Heat stress is an important abiotic stress, causing cellular damage including misfolding and aggregation of proteins, membrane damage, disruption of microtubule organization and accumulation of reactive oxygen species (ROS) (Rao et al., 2010; Mayer, 2012), eventually leading to arrested plant growth and development. To cope with heat stress, plants have evolved defense mechanisms including basal thermotolerance induced by a gradual increase to the normally lethal temperature and acquired thermotolerance induced by a short acclimation period at moderately high temperatures or other non-lethal stress prior to heatstress (Larkindale et al., 2005). During basal and acquired thermotolerance, genes encoding heat shock proteins (HSPs) are transcriptionally regulated by transcription factors including heat shock factors (HSFs) to protect plants against stress by re-establishing normal protein conformation and, thus, cellular homeostasis (Wang W. et al., 2004; Kotak et al., 2007; Mittler et al., 2012; Waters, 2013).

To respond effectively to heat stress, it is crucial for plants to sense the heat stress, which will translate the signals into the appropriate HSRs. Plants employ a set of sensors that perceive heat stress; these sensors include a plasma-membrane channel that initiates an inward calcium flux, a histone sensor in the nucleus, 2 unfolded protein sensors in the endoplasmic reticulum (ER) and the cytosol (Mittler et al., 2012), phytochrome B (Jung et al., 2016; Wang et al., 2017) and an ER-localized bZIP28 (Srivastava et al., 2014). The perception of heat stress by these sensors initiates complicated downstream signaling networks involving Ca^2+^ signaling (Delk et al., 2005; Saidi et al., 2011; Jia et al., 2014), phytohormones including salicylic acid (SA) (Clarke et al., 2004, 2009), jasmonic acid (JA) (Clarke et al., 2009), ethylene (ET) (Larkindale and Huang, 2004) and abscisic acid (ABA) (Larkindale and Huang, 2004; Halter et al., 2014), ROS such as hydrogen peroxide (Dat et al., 1998; Saidi et al., 2011), nitric oxide (Zhao et al., 2010; Wang L. et al., 2014), and the MAPK cascade (Zhai et al., 2017; Wang et al., 2018). These signaling pathways amplify signals and transduce them to the nucleus where many defense-related genes are transcriptionally modulated, leading to appropriate thermotolerance. This is regulated by various transcription factors (TFs) such as HSFs (Liu et al., 2013; Xue et al., 2015), SQUAMOSA PROMOTER BINDING PROTEIN-LIKE (Chao et al., 2017), WRKY (Dang et al., 2013), and NAC family members (Guan et al., 2014). These TFs are recruited and regulated by complicated signaling networks. The dissection of these signaling networks is an important approach to elucidating the mechanism underlying plant thermotolerance. However, the components of the signaling network during HSR have not been fully identified or characterized.

Phosphorylation that is mediated by protein kinases is an important regulatory mechanism in cellular signal transduction. Plant RLKs contain a cytoplasmic serine/threonine protein kinase domain, a single membrane-spanning segment and a large extracytoplasmic domain that can be divided into the S-domain class, leucine-rich repeat (LRR) class and a class that has epidermal growth factor-like repeats (Walker, 1994). Receptor-like kinases in plants are encoded by a big gene family, including at least 610 members in *Arabidopsis thaliana* and 1132 in rice (*Oryza sativa*) (Shiu et al., 2004). Leucine-rich repeat receptor-like kinases (LRR-RLKs) constitute the largest RLK subfamily with at least 223 members in Arabidopsis (Gou et al., 2010). They are characterized by 1-32 copies of leucine-rich repeats in their extracellular domains (Shiu and Bleecker, 2001a,b; Lehti-Shiu et al., 2009), and can be grouped into 13 subfamilies (LRR I to XIII) (Shiu and Bleecker, 2001a). Based on the presence or absence of an arginine aspartate (RD) motif in the kinase domains, they can be classified as RD-or non-RD LRR-RLKs (Wu et al., 2015). Many non-RD LRR-RLKs such as LRK10, FLS2 (Gómez-Gómez and Boller, 2002; Göhre et al., 2008), and Xa21 (da Silva et al., 2004) do not autophosphorylate the activation loop and act as potential pathogen/microbe-associated molecular pattern recognition receptors (Dardick and Ronald, 2006). RD-LRR RLKs such as SIRK, SYMRK, BAK1/SERK3, SCM, RKF1, PSK, Bri1/Systemin, WAKL, and ERECTA have been implicated in a broad spectrum of biological processes including plant growth, development, environmental stress response and plant immunity (Godiard et al., 2003; Dardick and Ronald, 2006; Sánchez-Rodríguez et al., 2009). In addition, two Arabidopsis RLKs, HAESA (HAE) and HAESA-like 2 (HSL2) play roles in the regulation of floral organ abscission (Cho et al., 2008; Stenvik et al., 2008; Patharkar and Walker, 2015; Baer et al., 2016). However, the results described above were largely drawn from a small numbers of RLKs in the model plants rice and Arabidopsis. The roles of RLKs in other non-model plants, particularly in thermotolerance, remain to be investigated.

Pepper is one of the most important vegetables worldwide. Exposure to frequent heat stress can cause damage to pepper plants, resulting in arrested growth, development and productivity. In particular, frequent concurrent exposure to high temperature and high humidity (HTHH) might leave pepper plants more susceptible to pathogens by direct damage, or by causing serious diseases via attenuating pepper immunity on the one hand and promoting the development of associated soil-borne pathogens on the other hand. *Ralstonia solanacearum* (*R. solanacearum*) is a typical soil-borne pathogen in pepper field, and it causes pepper bacterial wilt, one of the most important pepper diseases in worldwide. Our previous studies have revealed that CaWRKY6, CaWRKY40, CabZIP63, and CaCDPK15 act as positive regulators in the response of pepper to HTHH, with *CaWRKY40* directly regulated by CaWRKY6 and CabZIP63, and indirectly regulated by CaCDPK15, forming positive feedback loops (Wang et al., 2013; Cai et al., 2015; Shen et al., 2016a,b). In the present study, with an approach of gain- and loss-of-function assay by transient overexpression and virus induced gene silencing, respectively, we provided evidence that *CaHSL1* (HAESA-LIKE1 of *Capsicum annuum*), encoding an RD RLK, acts directly as a positive regulator in the thermotolerance of pepper under HTHH and is transcriptionally regulated by CaWRKY40, our result might benefit the elucidation of molecular mechanism underlying pepper thermotolerance.

## Materials and Methods

### Pepper and Tobacco Plant Cultivation and Heat Stress or Exogenous Hormone Treatments

Pepper plants (variety CM334) and *Nicotiana benthamiana* plants were cultivated using the method described in our previous study (Cheng et al., 2017). *R. solanacearum* strain FJC100301 (Dang et al., 2013) was cultured using a previously described method (Cheng et al., 2017). The bacterial cell solution used for inoculation was diluted to 10^8^ cfu mL^−1^ (OD_600_ = 0.8). For root inoculation, pepper and tobacco plants at the 8-leaf stage were irrigated with 1 mL of the resulting *R. solanacearum* suspension. For leaf inoculation, the third leaves from the top of the pepper or tobacco plants at the 8-leaf stage were infiltrated with 10 μL of the *R. solanacearum* suspension using a syringe without a needle, and the mock-treated control was inoculated with 10 mM MgCl_2_.

For the assay of the transcript levels of *CaHSL1*, the leaves of pepper plants at the 4-leaf stage were sprayed with 1 mM of SA (in 10% distilled ethanol), 100 μM of MeJA (in 10% distilled ethanol), 100 μM of ETH (in sterile double-distilled H_2_O [ddH_2_O]) or 200 μM of ABA (in sterile double-distilled H_2_O). The mock treatment was performed by spraying with a corresponding solvent or sterile ddH_2_O.

For HTHH, the *TRV::CaHSL1* and *TRV::00* and wild pepper plants were exposed to heat stress at 42°C or other temperatures as indicated under 90% humidity in darkness to exclude the effect of dehydration, and then were either harvested at indicated time points to isolate total RNA for assay of transcriptional levels of *CaHSL1*, or monitored for heat-stress damage.

### Vector Construction

All the vectors used in the present study were constructed using Gateway cloning technology (Invitrogen, Carlsbad, CA, United States). The full length ORF of *CaHSL1, CaWRKY40 or* (with or without the termination codon) were cloned to the entry vector pDONR207 by BP reaction, then to various destination vectors including pEarleyGate201, pEarleyGate103 (containing a GFP protein tag for subcellular localization) or pEarleyGate202 [containing a FLAG protein tag for chromatin immunoprecipitation (ChIP) analysis] by LR reaction using a gateway cloning technique (Invitrogen, Carlsbad, CA, United States). To construct the vector for VIGS to avoid possible off targeting, two specific fragments of *CaHSL1* were employed, one is 360 bps in length from the ORF and the other is 300 bps in length from the 3′ UTR of *CaHSL1*, to construct the vector for *CaWRKY40* silencing, a fragment of 300 bps in length from the 3′UTR of *CaWRKY40* were used. The sequence specificities of all of these fragments were further confirmed by searching with BLAST against genome sequence in database of CM334^1^ and Zunla-1^2^, which were cloned individually into the entry vector pDONR207, and then cloned into the PYL279 destination vector by BP and LR reaction.

### Virus Induced Gene Silencing (VIGS) of *CaHSL1* in Pepper Plants

For VIGS of *CaHSL1* in pepper plants, two specific fragments of 200–500 bps in length in the ORF or 3′ UTR were used to construct the VIGS vectors *TRV::CaHSL1-1* and *TRV::CaHSL1-2*, respectively, which were transformed into agrobacterium strain GV3101 cells. GV3101 cells containing PYL192 (TRV1) and *TRV::CaHSL1-1*, *TRV::CaHSL1-2* or *TRV::00* (as a negative control) were resuspended in the induction medium at 1:1 ratio (OD_600_ = 0.6) used in the VIGS following the method in our previous study (Dang et al., 2013), and were co-infiltrated into cotyledons of 2-week-old pepper plants.

### Transient Expression of *CaHSL1* or *CaWRKY40* in Pepper Leaves

For transient expression analysis, GV3101 cells containing the *35S::CaHSL1-HA* or *35S::CaWRKY40-HA*, *35S::HA* construct were grown overnight and resuspended in the induction medium (10 mM MES, 10 mM MgCl_2_, 200 μM acetosyringone, pH 5.6) to OD_600_ = 0.8, 10 μL of which was infiltrated into the leaves of pepper plants at the 8-leaf stage using a syringe without a needle. The injected leaves were monitored for phenotyping or collected at the indicated time points for further assays, including trypan blue and diaminobenzidine (DAB) staining or total RNA isolation.

### Subcellular Localization

GV3101 cells containing different vectors were grown overnight and then resuspended in the induction medium to OD_600_ = 0.8 for further use. To assay subcellular localization of CaHSL1 in epidermic cells of *Nicotiana benthamiana* leaves, GV3101 cells containing *35S::CaHSL1-YFP* (*35S::YFP*) were infiltrated into *Nicotiana benthamiana* leaves using a syringe without a needle, the inoculated leaves were harvested and the YFP signals in the in epidermic cells were observed using a Laser Scanning Confocal Microscope (TCS SP8, Leica, Solms, Germany) with an excitation wavelength of 513 and a 527 nm band-pass emission filter. For assay subcellular localization of CaHSL1 in pepper protoplasts, GF3101 cells containing *35S::CaHSL1-GFP* and GV3101 cells containing *35S:CBL1n-CFP* were mixed at a 1:1 ratio, the mixed cells were infiltrated into pepper leaves, which were harvested at 48 hpi and protoplasts were isolated following the method of Yoo et al. (2007), with a slight adjustment that 1.25% cellulase plus 0.3% macerozyme was employed. In order to burst the protoplasts, distilled water was added to the protoplasts suspension, GFP signals were monitored during protoplast rupture under Laser Scanning Confocal Microscope with an excitation wavelength of 488 nm and a 530 nm band-pass emission filter, while CFP signals were observed with a 434 and 477 nm band-pass emission filter.

To confirm the results of subcellular localization of CaHSL1 by detection of GFP, cytoplasmic protein and nuclear protein were isolated with specific kits from the pepper leaves transiently overexpressing CaHSL1-Flag, respectively. These protein extracts were incubated with anti-FLAG agarose (Thermo Fisher Scientific, Waltham, MA, United States) at 4°C overnight. Beads were collected and washed with Tris-buffered saline and Tween 20 (0.05%). The eluted protein was examined by immunoblotting with the help of anti-FLAG-peroxidase antibodies (Abcam, Cambridge, United Kingdom).

### Imaging-PAM

As high Fv/Fm (Marias et al., 2017) and actual photochemical efficiency of PSII in the light (ΦPS II) (Yan et al., 2008) were used as indicator of thermostability of plants, a MINI-version of the Imaging-PAM (Heinz Walz GmbH, Effeltrich, Germany) was used to image these two chlorophyll fluorescence parameters of *CaHSL1* silenced or *CaHSL1* transient overexpressing pepper leaves. The pepper plants were darkly adapted for 15 min and then directly put into the instrument for testing according to Schreiber et al. (1986) and Li et al. (2008).

### Histochemical Staining

Leaf staining with trypan blue and DAB was done according to a previously described method (Choi et al., 2012; Dang et al., 2013; Cai et al., 2015; Liu et al., 2015; Zhang et al., 2015).

### Immunoblotting

Total protein extracts were incubated with anti-GFP or anti-FLAG agarose (Thermo Fisher Scientific, Waltham, MA, United States) at 4°C overnight. Beads were collected and washed with Tris-buffered saline and Tween 20 (0.05%). The eluted protein was examined by immunoblotting with the help of anti-GFP-peroxidase or anti-FLAG-peroxidase antibodies (Abcam, Cambridge, United Kingdom).

### ChIP Analysis

ChIP analysis was performed following a previously described protocol (Ifnan et al., 2018). *CaWRKY40-HA* was transiently overexpressed in pepper leaves. The leaves were collected at 24 hpi and crosslinked with 1% of formaldehyde. The chromatin was isolated and sheared into fragments of 300–500 bp length, and the DNA-protein complexes were immunoprecipitated using anti-HA antibodies. The samples were de-crosslinked, and DNA was purified and employed as a template for PCR using specific primer pairs of the fragment containing W-box or fragment without W-box within the promoter of *CaHSL1* by semi-quantitative PCR using gene-specific primers (Supplementary Table S1).

### Quantitative Real-Time RT-PCR (qRT-PCR)

The qRT-PCR was used to detect the relative transcript levels of selected genes with specific primer pairs (Supplementary Table S1). A BIO-RAD Real-time PCR system (Foster City, CA, United States) and SYBR Premix Ex Taq II system (TaKaRa, Dalian, China) were used. Total RNA preparation and real-time RT-PCR were carried out as described in previous studies (Dang et al., 2013; Cai et al., 2015; Zhang et al., 2015). Five biological replicates of each treatment were performed. Data were analyzed by the Livak method (Livak and Schmittgen, 2001) and expressed as a normalized relative expression level (2^−ΔΔCT^) of the respective genes. The relative transcript level of each sample was normalized to *CaActin* (GQ339766) and 18S ribosomal RNA (EF564281).

## Results

### Cloning and Sequence Analysis of a Putative Receptor-Like Protein Kinase That Responds to Heat Stress

Since RLKs have seldom been characterized in the plant response to heat stress, we focused on a putative RLK in the present study because promoter scanning of genome sequence data^3^ with^4^ showed that its promoter region contains a putative heat-stress responsive heat shock element (HSE) and one W-box (Supplementary Figure S1C). The corresponding cDNA fragment containing a full-length open reading frame (ORF) was cloned by RT-PCR with a specific primer pair using the cDNA library derived from heat-stress challenged plants of the pepper line CM334 as a template. The amplified DNA fragment contained an ORF of 3096 bp, encoding a protein of 1031 amino acids.

The amino-acid sequence deduced by a SMART-analysis (Schultz et al., 2000) contains 5 LRR motifs, one transmembrane region and one Serine/Threonine protein kinase domain with the conserved G-T/S-XX-Y/F-X-APE motif (Krupa et al., 2004) that shared 100, 85, 65, 59, 47, and 41% sequence similarity to that in *Solanum tuberosum*, *Solanum lycopersicum*, *Nicotiana tabacum*, *Erythranthe guttata*, *Tarenaya hassleriana*, *Oryza sativa*, and *Arabidopsis thaliana*, respectively. As the corresponding protein in the majority of these plant species (including *Solanum tuberosum*, *Solanum lycopersicum*, and *Nicotiana tabacum*) was designated as HSL1 (HAESA-LIKE1), we named this gene *CaHSL1* (Supplementary Figures S1A,B). In addition, the Serine/Threonine protein kinase domain in CaHSL1 contains a conserved active-site signature motif (HRDVKSSNILLD) (Dardick et al., 2012), indicating that *CaHSL1* belongs to the RD RLK group.

### The Subcellular Localization of CaHSL1

**FIGURE 1:**
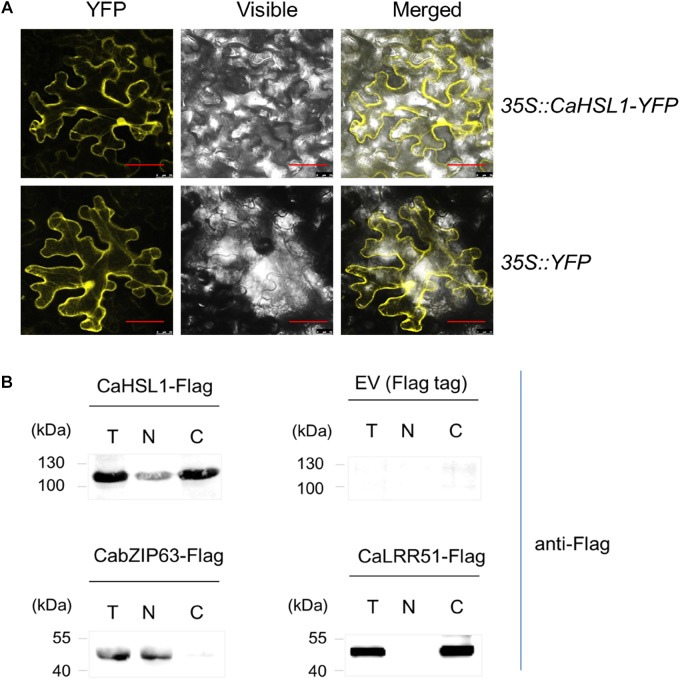
The subcellular localization of CaHSL1. **(A)** CaHSL1 was localized to the plasma membrane and nucleus when transiently overexpressed in leaves of *N. benthamiana* that were infiltrated with GV3101 cells containing *35S::CaHSL1-YFP* using *35S::YFP* as control. The Agrobacterium-infiltrated *N. benthamina* leaves were harvested at 48 hpi, and counterstained by DAPI. Images were taken by confocal microscopy. Control (*35S::YFP*) showed signal throughout the cell. Bars = 50 μm. **(B)** The detection of CaHSL1 in the nucleus and cytoplasm by immunoblotting with total (T), nuclear (N), and cytoplasmic (C) proteins isolated from *CaHSL1-FLAG* or FLAG transiently overexpressing *N. benthamina* leaves using antibodies to FLAG. α-FLAG, FLAG antibodies.

To determine the subcellular localization of CaHSL1 in epidermic cells of *Nicotiana benthamiana* leaves, CaHSL1-YFP was transiently overexpressed in *Nicotiana benthamiana* leaves, the YFP signals were observed in epidermic cells of *Nicotiana benthamiana* at 48 hpi, the result showed that YFP signals exhibited in the epidermic cells including plasma membrane, cytoplasm and the nuclei (Figure 1A). In parallel, the subcellular localization of CaHSL1 was assayed in pepper protoplast by transient overexpression of CaHSL1-GFP and we get similar result to that in epidermic cells of *Nicotiana benthamiana* leaves, and CaHSL1-GFP targeted to the plasma membrane similar to the fused CBL1n-CFP (as an additional control targeting to the plasma membrane) (Oliver et al., 2008) (Supplementary Figure S2A), indicating that CaHSL1 might localizes to the plasma membrane. To further determine whether CaHSL1 localize to plasma membrane, the subcellular localization of CaHSL1 was monitored at different time points in cracked pepper protoplasts by addition of 10 μL distilled water to 20 μL of CaHSL1-GFP or GFP overexpressing protoplasts suspension. The result showed that the GFP signals in CaHSL1-GFP overexpressing protoplasts were originally observed in a circular distribution in cell periphery (0 s), from 1.29 to 2.58 s, a considerable part of GFP signal in plasma membrane diminished, from 3.87 to 10.32 s, irregularly distributed GFP signal and spherical highly concentrated GFP signal, which might be invisible due to occlusion of cell membrane before 3.87 s, were observable. In the case of GFP overexpressing protoplasts, GFP signal was observed in the whole cells from 0 to 1.29 s, and GFP signal disappeared where the cell ruptured from 2.58 to 10.32 s and no spherical highly concentrated GFP signal was observed, indicating that GFP alone does not target to cell but that it is a soluble protein (Supplementary Figure S2B). A total of 5 protoplasts of CaHSL1-GFP or GFP overexpressing were monitored, and in all of them the GFP dynamic was identical. All these results indicate that CaHSL1 might localize to plasma membrane, cytoplasm and nuclei.

To confirm this result, fused *CaHSL1-FLAG* was transiently overexpressed by infiltrating *N. benthamiana* leaves with GV3101 cells containing *35S::CaHSL1-FLAG* using *35S::CabZIP63-FLAG* and *35S::CaLRR51-FLAG* as controls. The leaves were harvested at 24 hpi for nuclear and cytoplasmic protein isolation; the isolated proteins were also subjected to immunoblotting analysis using antibodies of FLAG. The results showed that the nuclear protein CabZIP63-FLAG, a protein targeted exclusively to the nucleus in our previous study (Shen et al., 2016b) and used as a control for nuclear targeting, was exclusively present in the nuclear protein fraction, whereas CaLRR51-FLAG [used as cytoplasmic targeting control which exclusively localizes to the plasma membrane by our previous study (Cheng et al., 2017)] was present only in the cytoplasmic protein fraction. The CaHSL1 protein was present in both nuclear and cytoplasmic fractions (Figure 1B). All these data suggest that CaHSL1 localize to plasma membrane, cytoplasm and nuclei, which is consistent to the presence of a TM and a NLS in the deduced amino acid sequence of CaHSL1.

### Transcript Levels of *CaHSL1* in Pepper Plants Upon Exposure to Heat Stress, *R. solanacearum* Inoculation, and Exogenous Applications of Phytohormones

**FIGURE 2:**
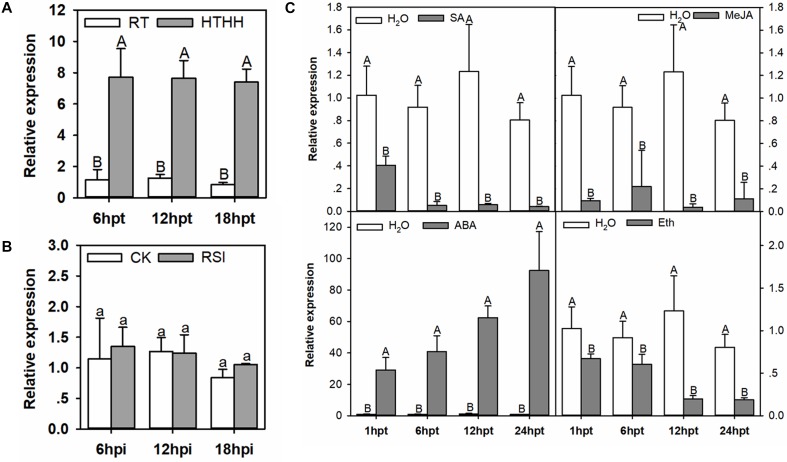
The qRT-PCR of *CaHSL1* transcript levels in pepper plants exposed to RSI, HTHH, and exogenous applications of SA, MeJA, ETH and ABA. **(A)** *CaHSL1* transcript levels at different time points in pepper leaves after HTHH (39°C, 90% humidity). **(B)** *CaHSL1* transcript levels measured at different time points in pepper leaves inoculated with the *R. solanacearum* strain FJC100301. **(C)** (top-left panel) transcript levels of *CaHSL1* in pepper plants treated with 1 mM SA at different time points; (top-right panel) transcript levels of *CaHSL1* in pepper plants treated with 100 μm MeJA at different time points; (bottom-left panel) transcript levels of *CaHSL1* in pepper plants treated with 100 μm ABA at different time points. (bottom-right panel) transcript levels of *CaHSL1* in pepper plants treated with 100 μm ETH at different time points. The transcript levels of *CaHSL1* in RSI, HTHH and SA, MeJA, ABA and ETH in pepper leaves were compared with those in mock-treated control plants, which were set to a relative expression level of “1.” Error bars indicate standard error. Data show the mean ± SD obtained from four independent experiments. Different upper-case letters indicate significant differences among means (*P* < 0.01), as calculated with Fisher’s protected-LSD-test. High temperature and high humidity treatment, HTHH; hpi, hours post infiltration; hpt, hours post treatment.

The presence of HSE and W-box in the promoter of *CaHSL1* implies its possible involvement in the response of the pepper plant to heat stress and pathogen infection, as WRKY proteins, which bind to W-boxes in promoters of their target genes, have been largely implicated in plant immunity (Eulgem et al., 2000; Zhang and Wang, 2005; Eulgem, 2006; Rushton et al., 2010) or heat stress responses (Li et al., 2009, 2010, 2011; Dang et al., 2013; Cai et al., 2015; He et al., 2016). To test this hypothesis, we used qRT-PCR to assay the transcript levels of *CaHSL1* in pepper plants challenged with *R. solanacearum* or heat stress under high humidity (42°C, 90% humidity, high humidity treatment is used to eliminate the effects of dehydration on plants during high temperature) compared to the in control plants. The transcript levels of *CaHSL1* were significantly higher in pepper plants challenged with HTHH for 6, 12, and 18 hpt (hours post-treatment), than in the plants kept at room temperature and high humidity, while transcript level of *CaHSL1* in pepper plants inoculated with *R. solanacearum* exhibited no significant difference from those in mock-treated plants (Figures 2A,B).

Phytohormones, such as SA, jasmonic acid (JA), ABA, and ET serve as important signaling molecules in the plant responses to pathogen or heat stress (Clarke et al., 2009; Huang et al., 2016; Suzuki et al., 2016; Verma et al., 2016). To test if the signaling pathways mediated by these molecules are involved in the regulation of *CaHSL1* expression, the transcript abundance of *CaHSL1* in pepper plants treated with SA, MeJA, ABA or ETH was measured against that in control plants. The results showed that transcript levels of *CaHSL1* were significantly down-regulated by exogenous applications of SA, MeJA, or ETH from 1 to 24 hpt but dramatically upregulated by exogenous ABA from 1 to 24 hpt (Figure 2C). These results suggest that *CaHSL1* might play a role in the response to heat stress.

Our data indicate that *CaHSL1* might participate in the response to heat stress but not to *R. solanacearum* inoculation in pepper.

### Silencing of *CaHSL1* Decreased Basal and Acquired Thermotolerance Under High Humidity in Pepper Plants but Did Not Alter Response to *R. solanacearum*

**FIGURE 3:**
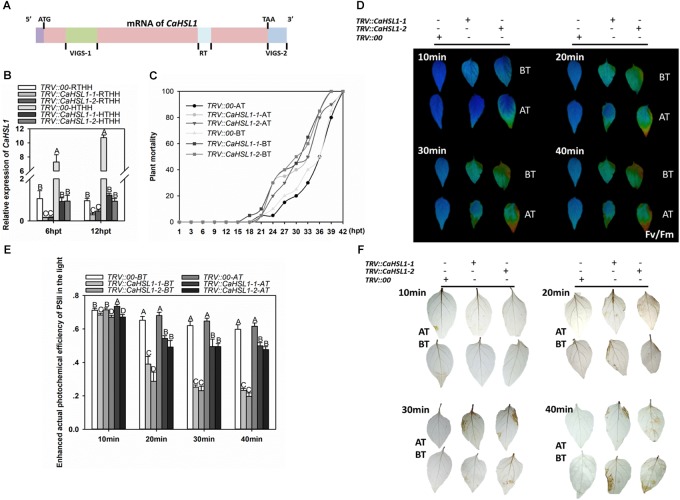
The effect of *CaHSL1* silencing by VIGS on response of pepper to HTHH. **(A)** Distribution of two specific fragments (VIGS-1 and VIGS-2) employed to construct the vectors (*TRV::CaHSL1-1* and *TRV::CaHSL1-2*) for *CaHSL1* silencing in pepper plants. **(B)** The transcript levels of *CaHSL1* in HTHH (42°C, 90% humidity) or RTHH (28°C, 90% humidity) treated *TRV::CaHSL1-1* and *CaHSL1-2* and *TRV::00* pepper plants by qRT-PCR, the transcript levels of *CaHSL1* in HTHH challenged *TRV::CaHSL1-1*, *TRV::CaHSL1-2* pepper plants were compared with those in mock-treated *TRV::00* plants, which were set to a relative expression level of “1.” **(C)** The plant mortality of *TRV::CaHSL1-1*, *TRV::CaHSL1-2* and *TRV:00* pepper plants challenged with HTHH (42°C, 90% humidity) pretreated with or without 37°C, a total of 50 plants of each genotypes were monitored from 1 to 42 hpt. **(D)** Fv/Fm shown in pseudo color images in HTHH challenged detached leaves of *TRV::CaHSL1-1*, *TRV::CaHSL1-2* and *TRV::00* plants pretreated with (AT) or without (BT) 37°C, which were detected immediately after HTHH treatment for 10–40 min. **(E)** ΦPSII in HTHH treated detached leaves of *TRV::CaHSL1-1, TRV::CaHSL1-2* and *TRV::00* plants pretreated with (AT) or without (BT) 37°C, which was detected immediately after HTHH treatment for 10, 20, 30, and 40 min. **(F)** The DAB staining of detached leaves of HTHH treated *TRV::CaHSL1-1, TRV::CaHSL1-2* and *TRV::00* plants pretreated with (AT) or without (BT) 37°C after the treatment of HTHH for 10 to 40 min. In **(B,C,E)**, Error bars indicate standard error, data show the mean ± SD obtained from four replicates. Different upper-case letters indicate significant differences among means (*P* < 0.01), as calculated with Fisher’s protected-LSD-test. hpt, hours post treatment. AT: acquired thermotolerance. BT: basal thermotolerance. Fv/Fm: the optimal/maximal photochemical efficiency of PSII in the dark; ΦPSII: the actual photochemical efficiency of PSII in the light.

With these *CaHSL1* silenced pepper plants, the effect of *CaHSL1* silencing on basal and acquired thermotolerance was determined (Figures 3A,B). To test the role of *CaHSL1* in pepper acquired thermotolerance, the plants were pretreated with 37°C under 90% humidity for 5 h, recovered for 24 h and then exposed to HTHH (42°C under 90% humidity), the mortality of HTHH challenged plants was calculated after 1–42 h of HTHH treatment, Fv/Fm (the optimal/maximal photochemical efficiency of PSII in the dark), an indicator of plant tolerance to heat stress (Yan et al., 2008; Wang Y. et al., 2014) and actual photochemical efficiency of PSII in the light (ΦPSII), an indicator of thermostability of the photosynthetic apparatus (Yan et al., 2008), were detected immediately after the HTHH treatment. The results showed that the *TRV::CaHSL1-1* and *TRV::CaHSL1-2* plants exhibited higher mortality from 1 to 42 hpt (Figure 3C), lower Fv/Fm shown in pseudo color in detach leaves or whole plants (Figure 3D and Supplementary Figure S3A) as well as lower ΦPSII in detach leaves or whole plants compared to that in the mock treated *TRV::00* plants (Figure 3E and Supplementary Figure S3B). To test if *CaHSL1* also play a role in pepper basal thermotolerance, pepper plants were HTHH and the mortality, Fv/Fm and ΦPSII of plants were measured as did in acquired thermotolerance assay, the result showed that *CaHSL1* silenced plants exhibited higher mortality, lower Fv/Fm and Φ_PSII_ after HTHH treatment in *CaHSL1* plants or their detached leaves compared to that in the mock treated wild type plants (Figures 3C–E and Supplementary Figure S2B). In addition, accumulation of H_2_O_2_, a typical ROS that causes peroxidative damage to plant tissues (RoyChoudhury et al., 2007; Chen et al., 2013), was measured in HTHH challenged plant leaves by staining with 3, 3′-Diaminobenzidine (DAB), the results showed that, compared to the mock treated *TRV::00* plants, darker DAB staining was detected in *TRV::CaHSL1-1* and *TRV::CaHSL1-2* plants after 10–40 min of HTHH treatment with or without 37°C pretreatment (Figure 3F). This result indicate that higher level of H_2_O_2_ accumulation and therefore probable heavier peroxidative damage might be caused by HTHH in *TRV::CaHSL1-1* and *TRV::CaHSL1-2* plants than in mock treated *TRV::00* plants. All these data suggest that silencing of *CaHSL1* significantly impaired pepper basal and acquired thermotolerance.

To further confirm the role of *CaHSL1* in pepper thermotolerance, the transcription of genes including *CaHSP24* (Guo et al., 2015a), *CaHSP24.2* (Guo et al., 2015a), *CaHSP70* (Guo et al., 2016), and *CaHsfA2* (Guo et al., 2015b) that act as positive regulators in pepper thermotolerance by previous studies was assayed by qRT-PCR, the results showed that HTHH-challenged *TRV::CaHSL1-1* and *TRV::CaHSL1-2* pepper plants with or without pretreatment with 37°C exhibited lower levels of *CaHSP24*, *CaHSP24.2*, *CaHSP70* and *CaHsfA2* transcript abundance at 6 and 12 hpt (Supplementary Figure S3C). All of these data supported that result that *CaHSL1* act as a positive regulator in pepper basal and acquired thermotolerance.

When the pepper plants were challenged with the *R. solanacearum* strain FJC100301, no significant difference in phenotype between *TRV::CaHSL1-1* and *TRV::CaHSL1-2* and *TRV::00* plants at 7 and 15 dpi was observed (data not shown), suggesting that the silencing of *CaHSL1* did not alter the response of pepper plants to *R. solanacearum* inoculation.

### Transient Overexpression of *CaHSL1* Induced Tolerance to Heat Stress and Upregulation of *CaHSP24* in Pepper Plants

**FIGURE 4:**
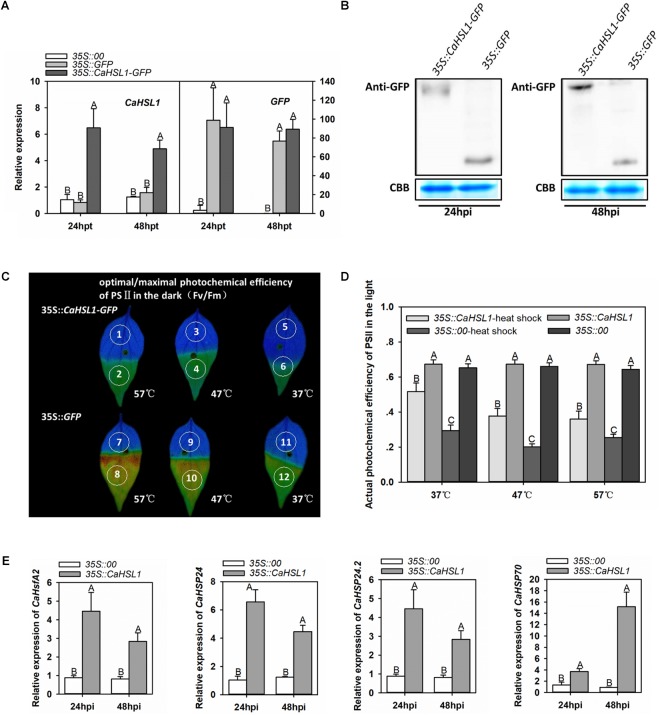
Transient overexpression of *CaHSL1* induced tolerance to HTHH and upregulation of thermotolerance associated genes in pepper plants. **(A)** The transcript levels of *CaHSL1* in *CaHSL1-GFP* transiently overexpressing pepper leaves by qRT-PCR. **(B)** The detection of transient expression of *CaHSL1-GFP* by immunoblotting using the protein isolated from pepper leaves infiltrated with GV3101 cells containing *35S::CaHSL1-GFP* with antibodies to GFP. **(C)** Fv/Fm shown in pseudo color images in *CaHSL1-GFP* transiently overexpressing pepper leaves challenged with HTHH compared to that in the mock treated control pepper leaves, the lower half leaf blades (1, 3, 5, 7, 9, 11) were kept in room temperature, while the upper half leaf blades 2 and 8, 4 and 10, 6 and 12 were inserted into water of 57, 47, and 37°C for 1 min, respectively, the pseudo color images were detected immediately after heat stress treatment. **(D)**ΦPSII in *CaHSL1-GFP* transiently overexpressing pepper leaves and the control plants challenged with (AT) or without (BT) HTHH, which were detected immediately after heat stress treatment by Imaging-PAM. **(E)** The transcript levels in *CaHSP24*, *CaHSP24.2*, *CaHSP70* and *CaHsfA2* in *CaHSL1-GFP* transiently overexpressing pepper leaves compared to the control plants. In **(A,E)**, the transcript levels of *CaHSL1*, *CaHSP24*, *CaHSP24.2*, *CaHSP70* or *CaHsfA2* in transiently *CaHSL1* overexpressing pepper leaves at different time points were compared to those in the mock treated control plants, which were set to a relative expression level of “1.” In **(A,D,E)**, error bars indicate standard error, data show the mean ± SD obtained from four replicates. Different upper-case letters indicate significant differences among means (*P* < 0.01), as calculated with Fisher’s protected-LSD-test. AT, acquired thermotolerance. BT, basal thermotolerance. hpi, hours post infiltration. hpt, hours post treatment. Fv/Fm, the optimal/maximal photochemical efficiency of PSII in the dark; ΦPSII, the actual photochemical efficiency of PSII in the light.

To confirm that *CaHSL1* acts as a positive regulator during pepper’s heat stress response, the function of *CaHSL1* in thermotolerance was further tested by transient overexpression in pepper leaves via infiltrating pepper leaves with *Agrobacterium tumefaciens* GV3101 cells carrying *35S::CaHSL1-GFP* (using *35S::GFP* as control). The success of *CaHSL1-GFP* transient overexpression was confirmed by qRT-PCR at transcriptional level (Figure 4A), and at posttranslational level by immunoblotting with antibodies against GFP 24 and 48 hpi (Figure 4B). The lower half blades of *CaHSL1-GFP* transiently overexpressing and the control pepper leaves were inserted into water of 57, 47, and 37°C for 1 min, and Fv/Fm shown in pseudo color images were measured after 15 min of darker adaptation, the result showed that the upper half blades of mock treated control pepper leaves exhibited a much brown color than that of the *CaHSL1-GFP* transiently overexpressing pepper leaves, although there was no difference in the lower half blades which was kept in the room temperature (Figure 4C), indicating a higher Fv/Fm in *CaHSL1-GFP* transiently overexpressing pepper leaves. In addition, the *CaHSL1-GFP* transiently overexpressing pepper leaves also exhibited higher ΦPSII than that of control plants upon the heat shock treatment for 1 min (Figure 4D). Furthermore, much higher levels of *CaHSP24*, *CaHSP24.2*, *CaHSP70* and *CaHsfA2* transcript were detected in *CaHSL1* transiently overexpressing pepper leaves than that in control leaves (Figure 4E). These results collectively suggest that the transient overexpression of *CaHSL1* enhanced thermotolerance probably through modulating the thermotolerance associated genes.

### CaWRKY40 Bind to the Promoter Region of *CaHSL1* Containing the W-box in Pepper Plants

**FIGURE 5:**
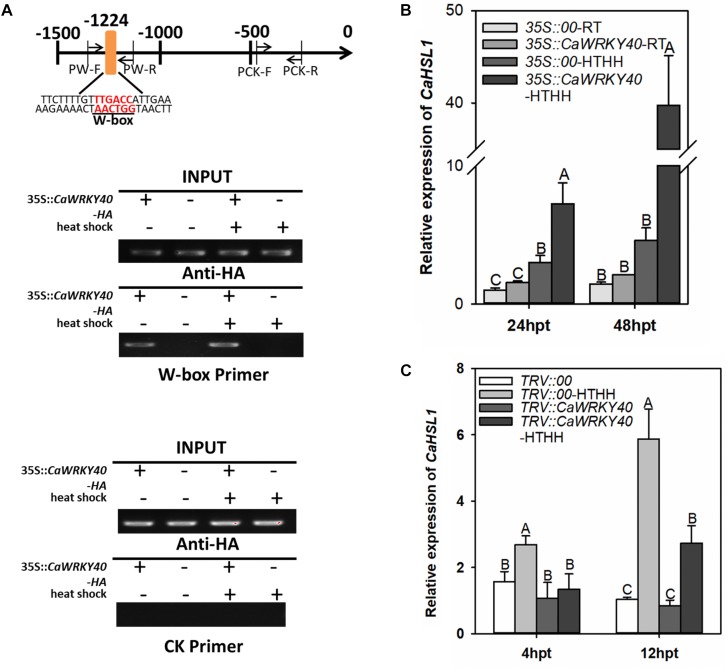
*CaHSL1* was directly regulated by CaWRKY40 at the transcriptional level. **(A)** ChIP assay showed that the W-boxes in the promoter of *CaHSL1* are bound by CaWRKY40. *CaWRKY40-HA* was transiently overexpressed in pepper leaves, the leaves were collected at 24 hpi and the DNA-protein complexes were immunoprecipitated using anti-HA antibodies. The immunoprecipitated DNA was employed as template for PCR using primer pairs specific to the fragment containing the W-boxes or without the W-box within the *CaHSL1* promoter. PW, specific primer pair for the fragment containing W-boxes within the promoter of *CaHSL*1; PCK, specific primer pair of the W-box free fragment within the promoter of *CaHSL1*. **(B)** Effect of transient overexpression of *CaWRKY40* on transcript levels of *CaHSL1* in pepper plants with or without HTHH challenge by qRT-PCR; **(C)** Effect of *CaWRKY40* silencing by VIGS in pepper leaves with or without HTHH challenge on transcription of *CaHSL1* by qRT-PCR. In **(B,C)**, *CaHSL1* transcript levels in *CaWRKY40* transient overexpressing or *CaWRKY40*-silenced pepper plants were compared with those in mock-treated control plants, which were set to a relative expression level of “1.” Data show the mean ± SD from four biological replicates; error bars indicate standard error. Different upper-case letters on the bars indicate significant differences between means (*p* < 0.01), as determined by Fisher’s protected LSD test.

Our previous study showed that CaWRKY40 acts as a positive regulator of the response of pepper to HTHH by binding to its target genes via a typical W-box in their promoters (Dang et al., 2013). Since only one W-box is present in the promoter of *CaHSL1*, it is possible that *CaHSL1* might be transcriptionally regulated by CaWRKY40 during the response of pepper to HTHH. We tested this possibility by ChIP-PCR using plants transiently overexpressing *CaWRKY40-GFP*. The enrichment of CaWRKY40 was found in the promoter of *CaHSL1* by PCR with DNA fragments precipitated from *CaWRKY40-GFP* overexpressing pepper leaves with antibodies of GFP as template using specific primer pair of the W-box containing fragment of *CaHSL1* promoter, whereas no binding signal was detected in the promoter fragment of *CaHSL1* that lacks a W-box (Figure 5A), indicating that CaWRKY40 binds to the promoter of *CaHSL1*, probably in a W-box-dependent manner.

### Transcription of *CaHSL1* Was Enhanced by Transient Overexpression of CaWRKY40 but Downregulated by Silencing of *CaWRKY40*

The direct binding of CaWRKY40 to the promoter of *CaHSL1* implies that *CaHSL1* might be transcriptionally regulated by CaWRKY40. To test this speculation, the effects of transient overexpression or silencing of *CaWRKY40* by VIGS were assayed (with or without HTHH) in pepper plants on transcription of *CaHSL1*. The transcript levels of *CaHSL1* were significantly enhanced in pepper leaves transiently overexpressing *CaWRKY40* compared to that in control plants (*35S::00*), regardless of HTHH challenge (Figure 5B). The silencing of *CaWRKY40* by VIGS was performed using a highly specific fragment within the 3′ UTR of *CaWRKY40*, the transcript level in HTHH treated *TRV::CaWRKY40* pepper plants was only 10–20% of that in the *TRV::00* plants, while no difference in the transcription of *CaWRKY40b*, a WRKY genes with the highest sequence similarity to *CaWRKY40* (Ifnan et al., 2018), was found between *TRV::CaWRKY40* and *TRV::00* plants was found by qRT-PCR (data not shown). The transcription abundance of *CaHSL1* was measured by qRT-PCR in HTHH challenged *TRV::CaWRKY40* and *TRV::00* plants, the result showed that transcript levels of *CaHSL1* were significantly lower in *TRV::CaWRKY40* than that in *TRV::00* plants at the two tested time points (Figure 5C). These results suggested that *CaHSL1* is directly and positively regulated by CaWRKY40 in pepper plants exposed to HTHH.

## Discussion

Although pepper is an important vegetable that suffers from heat stress, the molecular mechanism underlying pepper thermotolerance remains elusive. Our previous studies have shown that CaWRKY40 acts as a positive regulator in the response of pepper to HTHH or RSI (Dang et al., 2013; Cai et al., 2015; Shen et al., 2016a,b; Qiu et al., 2018). The present study builds on these data by demonstrating that *CaHSL1*, encoding an RD RLK, is target by CaWRKY40 and the CaHSL1 protein acts as a positive regulator in pepper basal and acquired thermotolerance under high humidity; however, CaHSL1 differs from CaWRKY40 in its role during the response of pepper to RSI.

**FIGURE 6:**
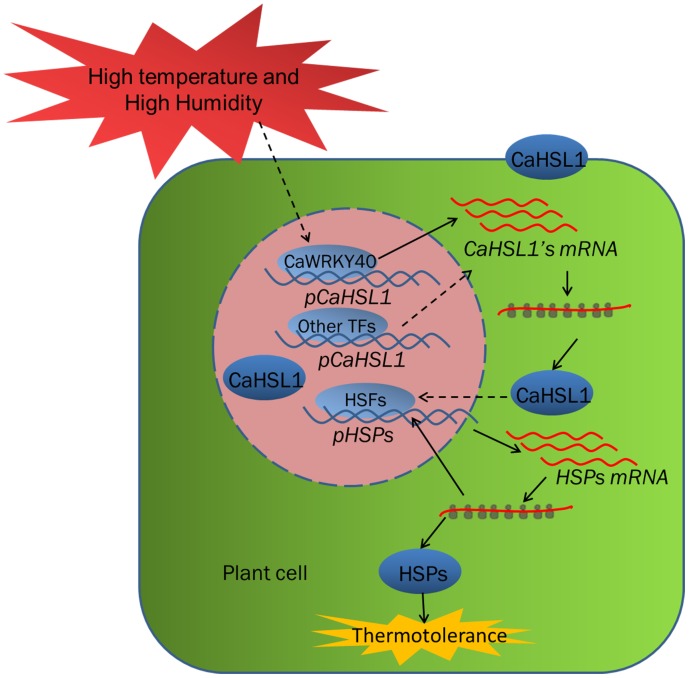
The relationship of the factors including CaHSL1 and CaWRKY40 under HTHH conditions. Upon the challenge of HTHH, *CaWRKY40* or other transcription factors such as HSFs are transcriptionally upregulated in some way, which in turn activate the transcription of *CaHSL1*. The upregulated CaHSL1 might positively modulate basal and acquired thermotolerance by activating the downstream signaling cascades. Solid line indicates direct effect, dotted lines indicate indirect or speculative relationships. HSFs, heat shock factors; HSPs, Heat shock proteins; HTHH, high temperature and high humidity.

The deduced amino-acid sequence of CaHSL1 shares domain-structure similarity with RLKs and high sequence similarity with putative HSLs from other plant species; it also contains a conserved arginine-aspartic acid motif in its S_TKc domain, thus suggesting that CaHSL1 belongs to the RD RLKs in pepper. *CaHSL1* exhibited transcriptional upregulation in pepper plants exposed to HTHH, *CaHSL1* silenced pepper plants by VIGS showed decreased both basal and acquired thermotolerance and optimal/maximal photochemical efficiency of PSII in the dark (Fv/Fm), and downregulation of the thermotolerance-associated gene *CaHSP24* (Heckathorn et al., 1996; Pivovarova et al., 2005). In contrast, transient overexpression of *CaHSL1* significantly enhanced the transcriptions of *CaHSP24*, *CaHSP24.2*, *CaHSP70* and *CaHsfA2* and significantly increased Fv/Fm as well as actual photochemical efficiency of PSII in the light, another indicator of thermal stability (Yan et al., 2008; Wang Y. et al., 2014) in pepper plants. These data collectively suggest a role for CaHSL1 as a positive regulator in pepper basal and acquired thermotolerance under high humidity. Similarly, previous studies have found that some regulatory proteins in HSR signaling, for example, AtHsfA3, Aldehyde dehydrogenases (ALDH) (Zhao et al., 2017), ATPase6 (ALA6) (Niu et al., 2017) HsfA2 (Ogawa et al., 2007) in Arabidopsis and LlHsfA3A in lily (*Lilium longiflorum*) (Wu et al., 2018), FaHsfA2c in *Festuca arundinacea* (Wang et al., 2017) act as positive regulator in both basal and acquired thermotolerance, while LlHsfA3B (Wu et al., 2018) in lily acts as positive regulator only in acquired thermotolerance, salicylic acid dependent signaling (Clarke et al., 2004) promotes only basal thermotolerance, indicating that basal and acquired thermotolerance might be modulated by different signaling pathways with extensive crosstalks between them. Noticeably, our data showed that *CaHSP24* and *CaHSP24.2* that might localize to chloroplast (Heckathorn et al., 1996) were dramatically downregulated by silencing of *CaHSL1*, coupled with significant decrease in Fv/Fm, whereas the transient overexpression of *CaHSL1* significantly enhanced transcription of *CaHSP24* and *CaHSP24.2* as well as enhanced Fv/Fm, it can be speculated that as an indicator of plant thermotolerance, the stability of photochemical efficiency of PSII (Fv/Fm) might be contributed by multiple HSPs including *CaHSP24* and *CaHSP24.2*, as Fv/Fm differed in timing and amplitudes with *CaHSP24* and *CaHSP24.2* during response *CaHSL1* silenced pepper plant to heat stress. *CaHSL1* might act as upstream components in HSR signaling and circuits the transcription of HSPs via various TFs including HSFs, as transcription of *CaHsfA2* was found to be modulated by CaHSL1 in the present study (Figure 6). However, the precise details how these HSPs are regulated by CaHSL1 and how they contribute to the stability of photochemical efficiency of PSII remain to be elucidated.

Abscisic acid functions as a key messenger in plant responses to biotic and abiotic stresses, including heat stress (Wang K. et al., 2014; Huang et al., 2016) and shows synergistic or antagonistic interactions with factors involved in immune responses (Moeder et al., 2010; Montillet and Hirt, 2013; Xu et al., 2013). The present study showed that *CaHSL1* was dramatically upregulated by exogenous ABA, further supporting the idea that *CaHSL1* acts as a positive regulator in the response of pepper to heat stress. Similarly, the RD RLK ERECTA act as a positive regulator in plant thermotolerance, as shown by gain- and loss-function analyses in Arabidopsis, rice and tomato (Shen et al., 2015). Another RLK, OsGIRL1, acts as a negative regulator in the response of Arabidopsis to heat stress (Park et al., 2014). Unlike ERECTA, which acts as positive regulator in plant immunity (ten Hove et al., 2011; Jordá et al., 2016), it appears that CaHSL1 does not play a role in pepper immunity, as the silencing of *CaHSL1* did not affect pepper responses to RSI. Not only was the expression of *CaHSL1* unaffected by RSI, it was downregulated by SA, MeJA or ETH, which activate the expression of immunity-associated genes and play important roles in the regulation of plant immunity (Dang et al., 2013, 2014; Cai et al., 2015; Shen et al., 2016a,b; Hussain et al., 2018; Ifnan et al., 2018; Qiu et al., 2018). We speculate that during infection by *R. solanacearum*, the transcription of *CaHSL1* and therefore the heat shock response mediated by CaHSL1 is blocked, which might benefit the recruitment of other resources to plant immunity. Furthermore, as heat-stress sensors targeting plasma membrane, endoplasmic reticulum, cytosol, and nucleus have been found (Mittler et al., 2012; Srivastava et al., 2014; Song et al., 2017), thus it puts forward the possibility that the whole-cell targeting of CaHSL1 might be required for signal transduction after perception of heat stress by the abovementioned sensors. However, the mechanisms for the initiation of CaHSL1-mediated HSR and the connection of CaHSL1 to these sensors remain to be elucidated.

Since thermotolerance consumes resources and energy, plants generally induce responses that minimize consumption of cellular resources. Genome-wide assays indicate that plants undergo massive reprogramming upon challenge by heat stress (Castandet et al., 2016; González-Schain et al., 2016; Liu et al., 2016; Gao et al., 2017); the transcriptional regulation of thermotolerance-associated genes by various transcription factors might play crucial roles in this process. *CaWRKY40*, which is transcriptionally modulated by CaWRKY6 (Cai et al., 2015) and CabZIP63 (Shen, et al., 2016a), acts as a positive regulator in the response of pepper to HTHH or RSI (Dang et al., 2013) by regulating different target genes (Cai et al., 2015; Shen et al., 2016a,b; Hussain et al., 2018; Ifnan et al., 2018; Qiu et al., 2018). Our data showed that the promoter of *CaHSL1* was bound by CaWRKY40; correspondingly, transcription of *CaHSL1* was positively regulated by CaWRKY40 during the response of pepper to heat stress but not to RSI, despite upregulation of *CaWRKY40* by RSI (Dang et al., 2013). Notably, the silencing of *CaWRKY40* in pepper plants did not abolish the upregulation of *CaHSL1* by HTHH, and a HSE is present in the promoter of *CaHSL1*, it can be speculated that the transcription of *CaHSL1* might also be modulated by unidentified HSFs, since HSFs have been suggested to participate in modulation of the expression of HSPs and other HS-induced transcripts during HSR by binding HSE in the promoters of their target genes (Kotak et al., 2007; Fragkostefanakis et al., 2015). It can also be speculated that the binding of *CaHSL1* promoter and transcriptional activation of *CaHSL1* by CaWRKY40 are modulated by unknown regulators specifically activated by heat stress but not by RSI, since the targeting and transcriptional activities of WRKY TFs have been found to be modulated by other regulators in protein-protein interactions (Chi et al., 2013). Further identification of these regulators might provide insight into the molecular mechanisms underlying thermotolerance mediated by CaHSL1. This non-linear relationship between different components of signaling pathways might provide plants with a powerful means to fine-tune their responses to be different environmental cues.

## Conclusion

Our data in the present study indicate that *CaHSL1* is transcriptionally upregulated by HTHH, leading to enhanced thermotolerance under high humidity. In addition, it was found that *CaHSL1* is transcriptionally regulated by CaWRKY40 directly during the response of pepper to HTHH. CaHSL1 might be used as potential target for the genetic improvement of pepper thermotolerance.

## Author Contributions

DG, FY, XX, YS, SY, and WC performed the experiments and analyzed the results. DG, FY, XX, and SH designed the experiments. SH wrote the manuscript.

## Conflict of Interest Statement

The authors declare that the research was conducted in the absence of any commercial or financial relationships that could be construed as a potential conflict of interest.

## Footnotes

1 http://peppergenome.snu.ac.kr/

2 http://peppersequence.genomics.cn/page/species/blast.jsp

3 http://peppergenome.snu.ac.kr

4 http://bioinformatics.psb.ugent.be/webtools/plantcare/html/

## Supplementary Material

FIGURE S1 The sequence analysis of CaHSL1 and *cis*-elements within the promoter of *CaHSL1*. **(A)** Schematic diagram of CaLRR-RLK1 protein domain architecture and multiple-sequence-alignment (MSA) of the amino-acid sequence of CaHSL1 with its homologues from other plant species; CaHSL1 shared 99, 75, 73, 72, 57, 61, 54, 49, and 48% sequence similarity to the sequences of HSL1s from *Capsicum baccatum*, *Capsicum chinense* and *Solanum lycopersicum*, *Solanum tuberosum*, *Nicotiana tabacum*, *Sesamum indicum*, *Coffea canephora*, *Glycine max*, *Brassica napus*, and *Arabidopsis thaliana*, respectively. Different colors indicate different percentages of similarity between CaHSL1 and HSL1s of the other species (i.e., black, red and green shades represent 100%, 75–100%, and 50–75% similarity, respectively). NLS: nuclear localization signal (Read bar in Supplementary Figure S1A); LRR, Leucine-rich repeats; TM, transmembrane region; S_TKc, Serine/Threonine protein kinases, catalytic domain. **(B)** Phylogenetic relationship between CaHSL1 and HSL1s of the above mentioned plants species. **(A,B)** were assayed using online SMART (http://smart.embl-heidelberg.de/smart/set_mode.cgi?NORMAL=1) and DNAMAN5 (Lynnon Biosoft, United States). **(C)** Distribution of stress responsiveness related *cis*-elements in the promoter of *CaHSL1* by PlantCARE (http://bioinformatics.psb.ugent.be/webtools/plantcare/html/), the “A” in the translational start codon ATG was set to +1. GARE, gibberellin-responsive element; W-box, TTGACC/T bound by WRKY TFs; HSE, *cis*-acting element involved in heat stress responsiveness; CGTCA, *cis*-acting regulatory element involved in MeJA-responsiveness; AuxRR, auxin-responsive region; LTR, low temperature-response element; TCA, *cis*-acting element involved in salicylic acid responsiveness.

FIGURE S2 Subcellular localization of CaHSL1 in protoplasts of pepper. **(A)** Subcellular localization of CaHSL1 in protoplasts of pepper, GV3101 cells containing *35S::CaHSL1-GFP* (*35S::GFP*) and *35S:CBL1n-CFP* were mixed at a 1:1 ratio and co-infiltrated into pepper leaves, which were harvested for protoplasts isolation, and the GFP signals were observed immediately. Bars = 50 μm. **(B)** Dynamics of the GFP fluorescence in protoplasts suspended in a hypotonic medium. Protoplasts were isolated from pepper leaves infiltrated with GV3101 cells containing *35S::CaHSL1-GFP* or *35S::GFP*, 10 μL of distilled water was added to a 20 μL of acquired protoplasts to rupture the protoplasts, and GFP signals were monitored using laser confocal at different time points after the addition of distilled water.

FIGURE S3 Chlorophyll fluorescence parameters and transcription of thermotolerance associated genes in *CaHSL1* silenced pepper plants upon the challenge of HTHH. **(A)** Phenotype and Fv/Fm shown in pseudo color images in detached leaves of *TRV::CaHSL1-1, TRV::CaHSL1-2* and *TRV::00* plants challenged with 42°C, 90% humidity pretreated with (AT) or without (BT) 37°C, the Phenotype and Fv/Fm shown in pseudo color images were measured at 1, 3, 12, and 24 hpt. **(B)** ΦPSII in HTHH challenged *CaHSL1* silenced pepper plants with (AT) or without (BT) 37°C compared to that in the mock treated wild type plants, which was detected 1, 3, 12, and 24 hpt. **(C)** The transcript levels of *CaHSP24*, *CaHSP24.2*, *CaHSP70* and *CaHsfA2* in HTHH challenged *TRV::CaHSL1-1,TRV::CaHSL1-1* pepper plants with (AT) or without (BT) pretreatment of 37°C compared with those in mock-treated *TRV::00* plants, which were set to a relative expression level of “1.” In **(B,C)**, error bars indicate standard error, data show the mean ± SD obtained from four replicates. Different upper-case letters indicate significant differences among means (*P* < 0.01), as calculated with Fisher’s protected-LSD-test. AT, acquired thermotolerance. BT, basal thermotolerance. HTHH, High temperature and high humidity treatment. hpt, hours post treatment. Fv/Fm, the optimal/maximal photochemical efficiency of PSII in the dark. ΦPSII, the actual photochemical efficiency of PSII in the light.

TABLE S1 The primers used in PCR assay in the present study.
