# Supplementary figures and images for: CaHSL1 Acts as a Positive Regulator of Pepper Thermotolerance Under High Humidity and Is Transcriptionally Modulated by CaWRKY40
Source: Front Plant Sci. 2018 Dec 7;9:1802. doi: 10.3389/fpls.2018.01802 (PMC6292930; doi:10.3389/fpls.2018.01802)

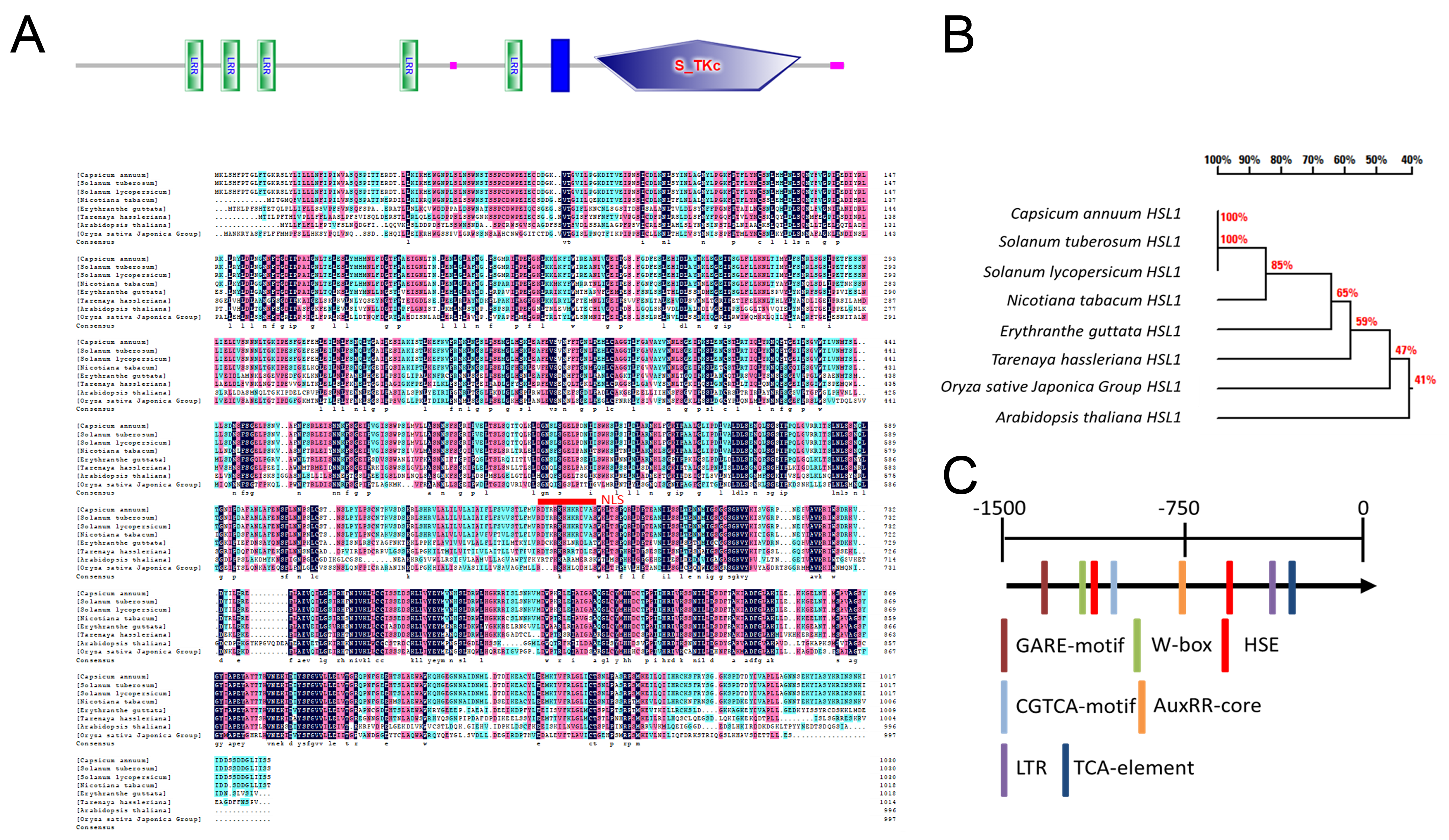

Supplement: FIGURE S1 — The sequence analysis of CaHSL1 and cis-elements within the promoter of CaHSL1. (A) Schematic diagram of CaLRR-RLK1 protein domain architecture and multiple-sequence-alignment (MSA) of the amino-acid sequence of CaHSL1 with its homologues from other plant species; CaHSL1 shared 99, 75, 73, 72, 57, 61, 54, 49, and 48% sequence similarity to the sequences of HSL1s from Capsicum baccatum, Capsicum chinense and Solanum lycopersicum, Solanum tuberosum, Nicotiana tabacum, Sesamum indicum, Coffea canephora, Glycine max, Brassica napus, and Arabidopsis thaliana, respectively. Different colors indicate different percentages of similarity between CaHSL1 and HSL1s of the other species (i.e., black, red and green shades represent 100%, 75–100%, and 50–75% similarity, respectively). NLS: nuclear localization signal (Read bar in Supplementary Figure S1A); LRR, Leucine-rich repeats; TM, transmembrane region; S_TKc, Serine/Threonine protein kinases, catalytic domain. (B) Phylogenetic relationship between CaHSL1 and HSL1s of the above mentioned plants species. (A,B) were assayed using online SMART (http://smart.embl-heidelberg.de/smart/set_mode.cgi?NORMAL=1) and DNAMAN5 (Lynnon Biosoft, United States). (C) Distribution of stress responsiveness related cis-elements in the promoter of CaHSL1 by PlantCARE (http://bioinformatics.psb.ugent.be/webtools/plantcare/html/), the “A” in the translational start codon ATG was set to +1. GARE, gibberellin-responsive element; W-box, TTGACC/T bound by WRKY TFs; HSE, cis-acting element involved in heat stress responsiveness; CGTCA, cis-acting regulatory element involved in MeJA-responsiveness; AuxRR, auxin-responsive region; LTR, low temperature-response element; TCA, cis-acting element involved in salicylic acid responsiveness. [file Image_1.TIF]

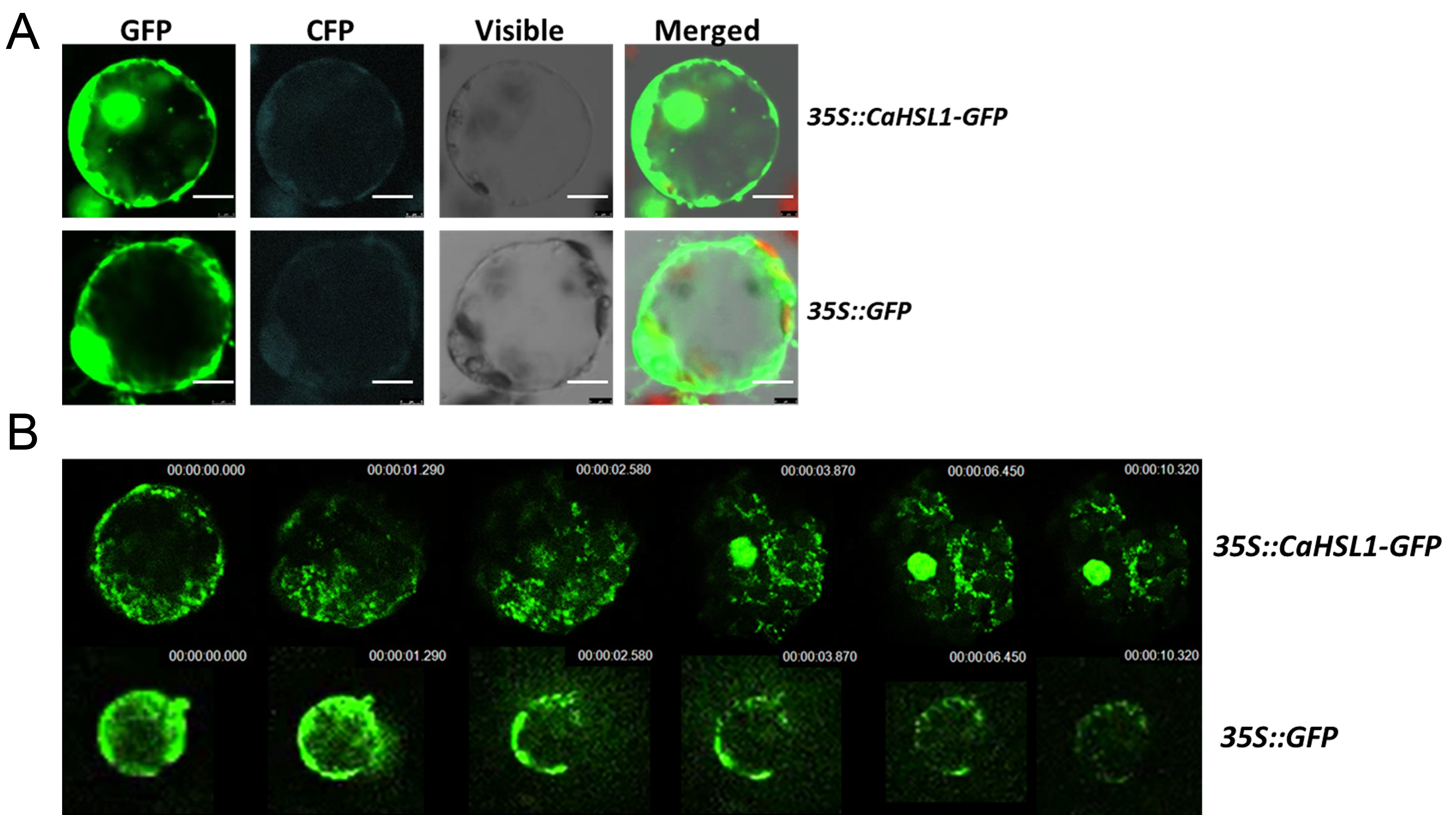

Supplement: FIGURE S2 — Subcellular localization of CaHSL1 in protoplasts of pepper. (A) Subcellular localization of CaHSL1 in protoplasts of pepper, GV3101 cells containing 35S::CaHSL1-GFP (35S::GFP) and 35S:CBL1n-CFP were mixed at a 1:1 ratio and co-infiltrated into pepper leaves, which were harvested for protoplasts isolation, and the GFP signals were observed immediately. Bars = 50 μm. (B) Dynamics of the GFP fluorescence in protoplasts suspended in a hypotonic medium. Protoplasts were isolated from pepper leaves infiltrated with GV3101 cells containing 35S::CaHSL1-GFP or 35S::GFP, 10 μL of distilled water was added to a 20 μL of acquired protoplasts to rupture the protoplasts, and GFP signals were monitored using laser confocal at different time points after the addition of distilled water. [file Image_2.TIF]

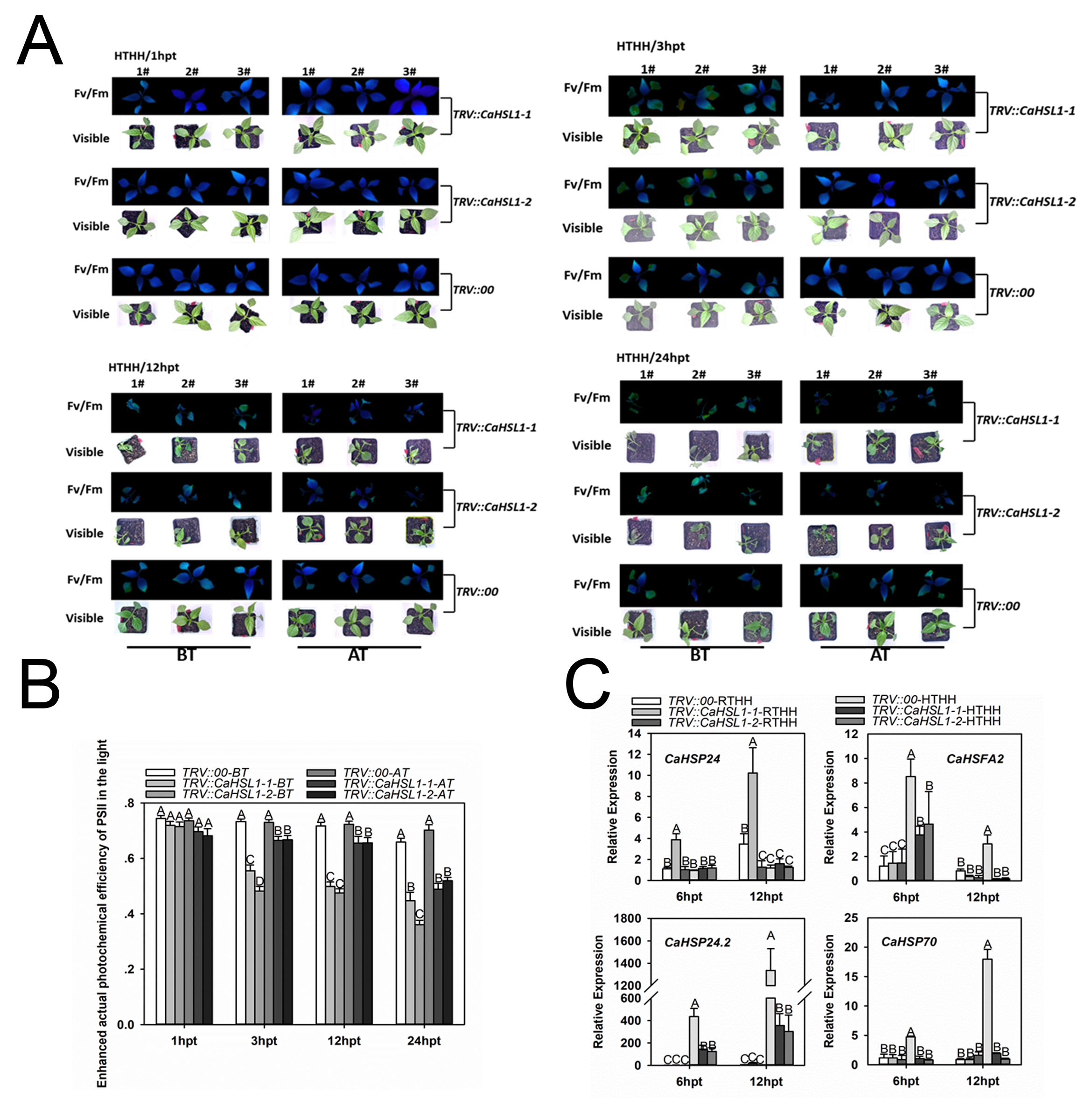

Supplement: FIGURE S3 — Chlorophyll fluorescence parameters and transcription of thermotolerance associated genes in CaHSL1 silenced pepper plants upon the challenge of HTHH. (A) Phenotype and Fv/Fm shown in pseudo color images in detached leaves of TRV::CaHSL1-1, TRV::CaHSL1-2 and TRV::00 plants challenged with 42°C, 90% humidity pretreated with (AT) or without (BT) 37°C, the Phenotype and Fv/Fm shown in pseudo color images were measured at 1, 3, 12, and 24 hpt. (B) ΦPSII in HTHH challenged CaHSL1 silenced pepper plants with (AT) or without (BT) 37°C compared to that in the mock treated wild type plants, which was detected 1, 3, 12, and 24 hpt. (C) The transcript levels of CaHSP24, CaHSP24.2, CaHSP70 and CaHsfA2 in HTHH challenged TRV::CaHSL1-1,TRV::CaHSL1-1 pepper plants with (AT) or without (BT) pretreatment of 37°C compared with those in mock-treated TRV::00 plants, which were set to a relative expression level of “1.” In (B,C), error bars indicate standard error, data show the mean ± SD obtained from four replicates. Different upper-case letters indicate significant differences among means (P < 0.01), as calculated with Fisher’s protected-LSD-test. AT, acquired thermotolerance. BT, basal thermotolerance. HTHH, High temperature and high humidity treatment. hpt, hours post treatment. Fv/Fm, the optimal/maximal photochemical efficiency of PSII in the dark. ΦPSII, the actual photochemical efficiency of PSII in the light. [file Image_3.TIF]
