# Supplementary material for: CaHSL1 Acts as a Positive Regulator of Pepper Thermotolerance Under High Humidity and Is Transcriptionally Modulated by CaWRKY40
Source: Front Plant Sci. 2018 Dec 7;9:1802. doi: 10.3389/fpls.2018.01802 (PMC6292930; doi:10.3389/fpls.2018.01802)
Supplement: TABLE S1 — The primers used in PCR assay in the present study. [file Table_1.DOCX]

**Table. S1** Primers for PCR and qRT-PCR used in this study.

| Gene | Accession no. | Forward primers (5’→3’) | Reverse primers(5’→3’) |
| --- | --- | --- | --- |
| Primers used for construction of over-expression vectors | | | |
| *CaHSL1-OE* |  | 5-GGGGACAAGTTTGTACAAAAAAGCAGGCTTC ATGAAGTTATCCCATTTTCC-3 | 5-GGGGACCACTTTGTACAAGAAAGCTGGGTC TTACACACTGGAGATTATTA-3 |
| Primers used for construction of subcellular localization vectors | | | |
| ΔTAA- *CaHSL1-OE* |  | 5-GGGGACAAGTTTGTACAAAAAAGCAGGCTTC ATGAAGTTATCCCATTTTCC-3 | 5-GGGGACCACTTTGTACAAGAAAGCTGGGTC CACACTGGAGATTATTA-3 |
| Primers used for construction of VIGS vector | | | |
| *CaHSL1-VIGS1* |  | GGGGACAAGTTTGTACAAAAAAGCAGGCTTC CACAGAATTACTTTGTGGGTC | GGGGACCACTTTGTACAAGAAAGCTGGGTC CGAAAGCCGATTACTGAA |
| *CaHSL1-VIGS2* |  | GGGGACAAGTTTGTACAAAAAAGCAGGCTTC TATTGGAGATAGGCAGAAAG | GGGGACCACTTTGTACAAGAAAGCTGGGTC TATCAACCAAAGGTTAACTC |
| Primers used for qRT-PCR analyses in pepper plants | | | |
| *CaHSL1*-RT |  | GGATTTGGAGCGATAGGA | GCGATGAATGATTGGTGG |
| *GFP* |  | ATCATGGCCGACAAGCAGAA | TCTCGTTGGGGTCTTTGCTC |
| *CaHSP24* | HM132040 | GTTCGTCTAGCAGTTTGGTTCGGTTG | GTAATTTAACTAAACAGACTCTTACAACC |
| *CaWRKY40* | AAX20040.1 | AACTTGGATGTTGTGCCTGGA | CTGTAACCTTGGCTTTTATGTGC |
| *CaHsfA2* |  | GTAGCATCAGTAGCCACAGC | CAAGCAACTCTTCCCAAATA |
| *CaHSP24.2* |  | CTTGCTCTTAGGAGGGCTACC | TCTGAGTGTTAGTATTGAAGGATCG |
| *CaHSP70* |  | GTGAGAATGTATGCCTGCTA | CCTGGTACTTGATTGCTGAT |
| *CaActin* | AY572427 | AGGGATGGGTCAAAAGGATGC | GAGACAACACCGCCTGAATAGC |
| Primers used for ChIP-qRT-PCR analyses in pepper plants | | | |
| *CaHSL1-Wbox* |  | CTGTTGAAGCTTCCATTG | CTGAGCTCCTCGAGC |
| *CK* |  | AATGGGATTTTACATTCA | AATAATCATTGAAAAT |
